# Two novel bacteriophage genera from a groundwater reservoir highlight subsurface environments as underexplored biotopes in bacteriophage ecology

**DOI:** 10.1038/s41598-020-68389-1

**Published:** 2020-07-17

**Authors:** Ole Hylling, Alexander B. Carstens, Witold Kot, Martin Hansen, Horst Neve, Charles M. A. P. Franz, Anders Johansen, Lea Ellegaard-Jensen, Lars H. Hansen

**Affiliations:** 1 Department of Environmental Science, Aarhus University, Frederiksborgvej 399, 4000 Roskilde, Denmark; 2 Department of Plant- and Environmental Sciences, Section for Microbial Ecology and Biotechnology, University of Copenhagen, Copenhagen, Denmark; 3 Department of Microbiology and Biotechnology, Max Rubner-Institut, Hermann-Weigmann-Straße 1, 24103 Kiel, Germany

**Keywords:** Ecology, Bacteriophages, Environmental microbiology, Phage biology, Microbiology, Microbial genetics, Viral genetics

## Abstract

Although bacteriophages are central entities in bacterial ecology and population dynamics, there is currently no literature on the genomes of bacteriophages isolated from groundwater. Using a collection of bacterial isolates from an aquifer as hosts, this study isolated, sequenced and characterised two bacteriophages native to the groundwater reservoir. Host phylogenetic analyses revealed that the phages targeted *B. mycoides* and a novel *Pseudomonas* species. These results suggest that both bacteriophages represent new genera, highlighting that groundwater reservoirs, and probably other subsurface environments as well, are underexplored biotopes in terms of the presence and ecology of bacteriophages.

## Introduction

Despite metagenomics revealing that groundwater reservoirs harbour complex bacterial communities that are closely associated with biogeochemical cycling, much remains uncharted about their microbial ecology^1^. In this context, it could be argued that bacteriophages (phages) have been studied to an even lesser extent, but they are generally considered to play a fundamental role in shaping bacterial communities and consequently influencing biogeochemical cycling^2, 3^. While viruses (including phages) only constitute 0.04% of the earth’s biomass^4^, they are widely regarded to constitute the largest and most diverse family of biological entities^5^. Thus, mapping out their taxonomy, distribution and ecological role is a daunting task. Currently, there are no genome sequences of phages, isolated from groundwater systems, despite descriptions of phage abundances and viromes in these systems^6,7^. This study isolated, sequenced and characterised two novel phages from a groundwater reservoir. To the authors’ knowledge, this is the first report on groundwater phages that includes their sequenced genome and phylogenomic affiliation. The aim of the present study was to describe the first isolated groundwater bacteriophages that target actual bacterial isolates from the groundwater reservoir through isolation, genome sequencing, bioinformatics and protein characterisation. Predator–prey pairs are of relevance not only in the study of environmental microbiology, including the food webs of groundwater aquifers, but also in the context of bioaugmentation for purification of contaminated groundwater, where knowledge of indigenous enemies is crucial to the survival of introduced degrader bacteria.

## Results and discussion

### Phage isolation and phage host identification

Using a collection of natural bacterial groundwater isolates as hosts, two phages—Anath (Genbank accession MG983742.1) and Lana (Genbank accession MK473373.1)—were successfully obtained and their hosts identified as *Bacillus mycoides* and *Pseudomonas* sp., respectively, by means of the publicly available online tool for strain identification Type (Strain) Genome Server (TYGS) (https://www.tygs.dsmz.de)^8^. Besides being the first sequenced groundwater phages, this is also the first record of a *B. mycoides* phage (https://www.millardlab.org; Millard Lab Bioinformatics—July 2019). Whole genome and 16S rRNA-based phylogenetic trees of the phages’ hosts, built in TYGS, are provided in Figures S1-S4 in the Supplementary Information.

### Phage morphologies

**Figure 1:**
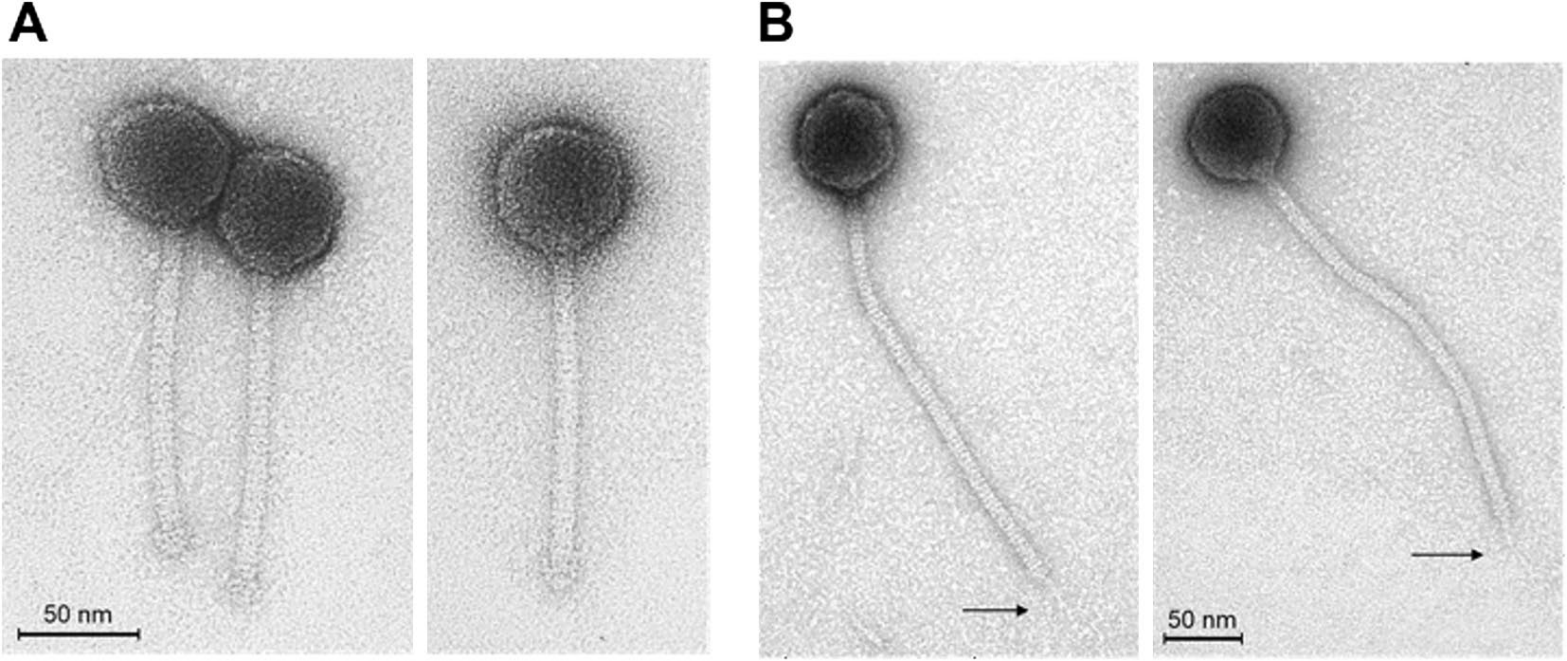
Transmission electron micrograph showing the long-tailed *Siphoviridae* morphotype of phage Anath (**A**) and phage Lana (**B**). The arrows indicate faint central tail-fibre structures.

Transmission electron microscopy of both phages (Fig. 1A,B) revealed morphologies to suggest that both Anath and Lana are affiliated to the family *Siphoviridae* in the order *Caudovirales*. This suggestion is also supported by the BLASTn results (described below). Phage Anath (Fig. 1A) exhibited a head diameter of 58 ± 2 nm and a tail length of 137 ± 3 nm, with the tail ending in a distinct baseplate structure (nine phage particles measured). Phage Lana (Fig. 1B) exhibited a head diameter of 67 ± 2 nm and a tail length of 275 ± 5 nm, with its tail ending with a thin central tail-fibre (106 ± 16 nm long) (25 phage particles measured).

### Burst size

For each phage, a one-step growth curve experiment^9^ was conducted to determine the phage latency period and phage burst size. A preliminary requirement to this experiment, though, is to establish the CFU–OD_600nm_ relationship of the host at the relevant conditions. This relationship is in turn used to determine the OD at which the appropriate concentration of (viable) host cells is reached, and a sensible inoculum is used for the given experimental conditions. Unfortunately, a reliable OD_600nm_–CFU relationship for the host of Anath was not possible due to the rhizoid growth behaviour of *B. mycoides*. Furthermore, microscopy observations during exponential growth of this host (data not shown) revealed that the cells existed not as singular cells, but predominantly as multicellular filamentous growth as well as singular cells. Naturally, this type of growth distorts an accurate burst size determination for Anath and stresses the need for the development of new methods to study phage-host interactions in non-model organisms. Thus, it was only possible to complete the one-step growth curve for Lana. For Lana, the latency period was found to be 106.67 ± 3.33 (SEM) min and burst size was determined to be 19.85 ± 3.55 (SEM) phage progenies per cell. The one-step growth curve for phage Lana is provided in Figures S5 in the Supplementary Information. The phage-host interactions were all performed under standard laboratory conditions (planktonic cells and room temperature). This evidently does not reflect the natural conditions in groundwater and as such it is possible that the growth parameters determined for Lana are not representative of its natural growth. Ideally, methods should also be developed that allow the study of phage-host interactions under conditions closer to those found in groundwater, such as cells growing in biofilms.

### General features of phage genomes

**Figure 2:**
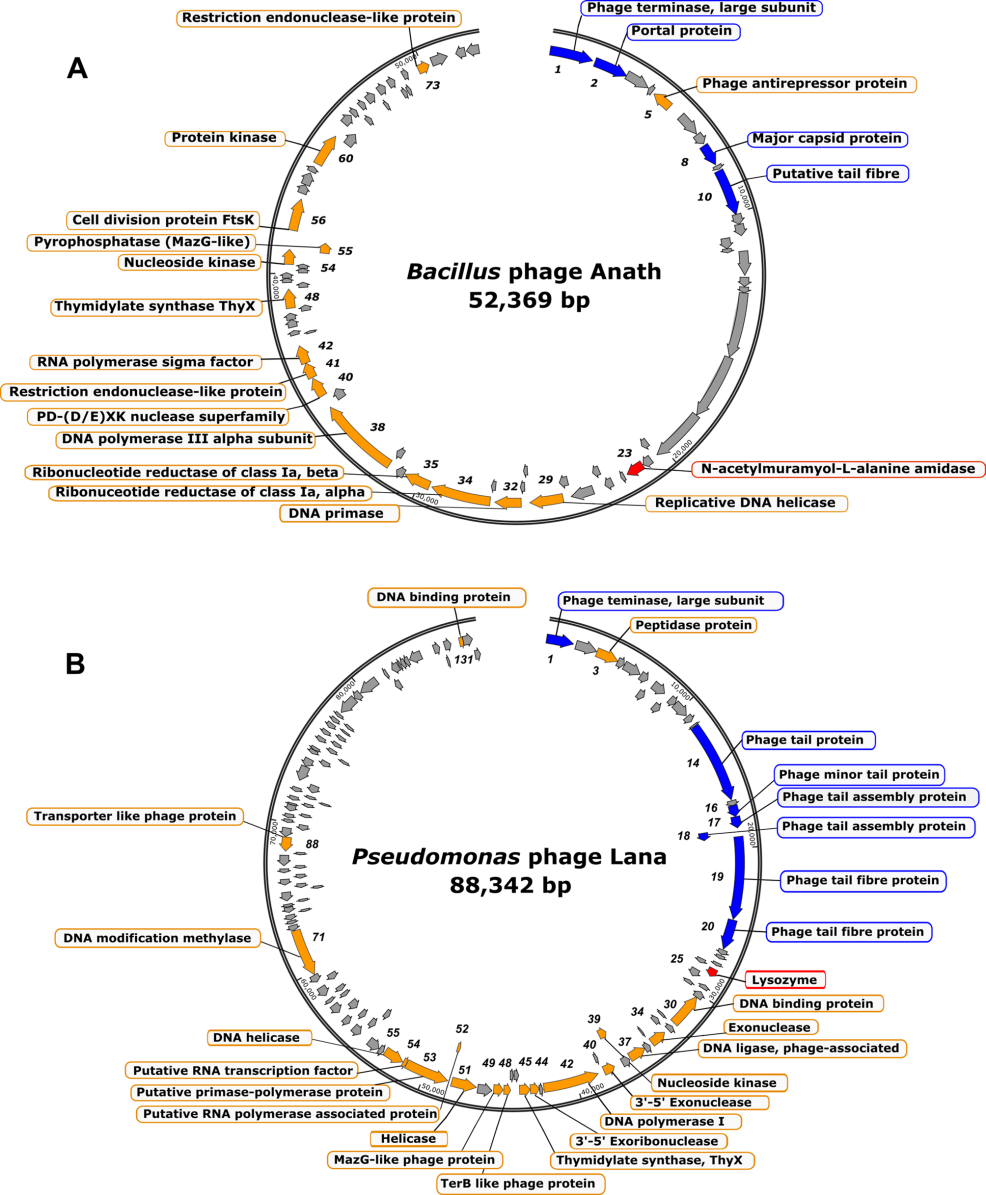
Genome structure of phage Anath (**A**) and phage Lana (**B**) with annotated putative protein functions (outside) and their gene product no. (inside). Structural genes are labelled blue and the lytic gene in red. Genes encoding hypothetical proteins are shown as grey.

Assembly of Anath (average coverage × 3,168) revealed a 52 369 bp linear genome with a 41.1% GC content. Interestingly, this is a higher GC content compared with its host (35.2%). Thus, the sequence of Anath is in contrast to the common trend found among phages that shows a lower^10,11^ or similar GC content to their hosts^12^, but falls within the variability of the phage-host GC ratio at this genome size^13^. Anath harboured 76 ORFs (Fig. 2A), and via protein database analysis, putative protein functions were assigned to 20 of the 76 ORFs. Furthermore, its genome contained genes with a similarity to counterparts found in other *Bacillus* phages^14^ associated with DNA replication/metabolism and lysis. An overview of phage Anath genes annotated with predicted functions is provided in Supplementary Table S1.

The assembly of phage Lana (average coverage × 142.6) revealed a linear 88 342 bp genome with a 60.8% GC content. The GC content of Lana was similar to its host (60.65%) and thus shared the common trend in phage-host GC relationships^13^. Putative protein functions were assigned to 27 of the 133 ORFs (Fig. 2B). An overview of phage Lana genes annotated with predicted functions is provided in Supplementary Table S2.

### Similarity with other phages

**Figure 3:**
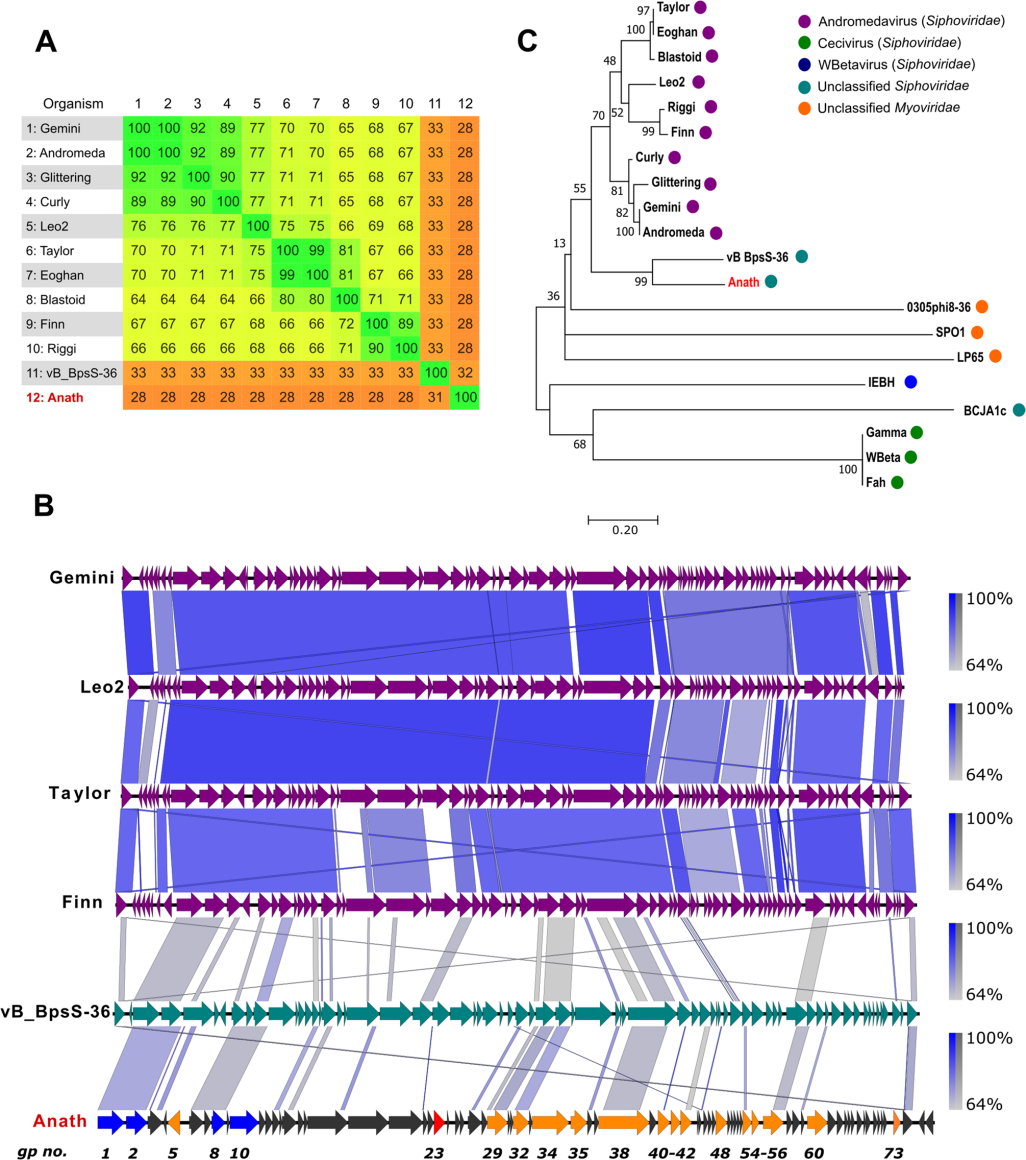
Comparative genomics and phylogenomic affiliation of phage Anath with other *Bacillus* phages. (**A**) Heat-map of fragmented multiple alignment (tBLASTx) constructed in Gegenees version 2.2.1 (accurate mode: fragment size = 200 bp, sliding-step size = 100 bp) between Anath (bold) and the 11 phages hits from BLASTn search. (**B**) Genome comparison of Anath (bottom) and selected representatives (Gemini, Leo2, Taylor, Finn and vB_BpsB-36), using the Easyfig genome visualisation tool in BLASTn mode (version 2.2.3). Nucleotide similarity at different regions between genomes is given in percentages (right bar) (**C**) Maximum-likelihood phylogram, based on the terminase nucleotide sequence of Anath (highlighted as red), vB_BpsS-36 the Andromedavirus genus and eight outliers. The selected phages are labelled according to their taxonomy classification in GenBank (right bar). The percentage of trees in which the associated taxa clustered together is shown next to the branches. A bootstrap value of 100 was used in the analysis.

As one of the first two sequenced groundwater phages and a novel *B. mycoides* phage, Anath was expected to show a weak resemblance to known phages. A BLASTn search^15^ of Anath (June 2019) resulted in 11 *Siphoviridae* hits, of which one was an unclassified *Siphoviridae* member—vB_BpsS-36 (Genbank accession no. MH884513.1, 22% coverage, ~ 70% sequence identity)—and ten belonged to the genus *Andromedavirus* (10–15% coverage, ~ 70% sequence identity); Curly (Genbank accession no. KC330679.1), Andromeda (Genbank accession KC330684.1), Gemini (Genbank accession KC330681.1), Glittering (Genbank accession KF669651.1), Leo2 (Genbank accession KU836751.1), Finn (Genbank accession KC330683.1), Taylor (Genbank accession KC330682.1), Eoghan (Genbank accession KC330680.1), Riggi (Genbank accession KF669659.1) and Blastoid (Genbank accession KF669648.1). An overview of BLASTn results, E-value, query coverage and sequence identity is provided in Supplementary Table S3. The phylogenomic relationship between Anath and these phages was analysed using Gegenees (version 2.2.1)^16^ in a tBLASTx fragmented multiple alignment (accurate mode; fragment size = 200 bp, sliding-step size = 100 bp). The analysis revealed a similarity in amino acid sequence of just 28–32% between Anath and any of the included phages (Fig. 3A). Genomes were then compared between Anath, vB_BpsS-36 and selected representatives of *Andromedavirus* (Gemini, Leo2, Taylor and Finn) using the Easyfig visualisation tool (version 2.2.3)^17^ in BLASTn mode (Fig. 3B). As expected, the results revealed similar synteny with the closely related *Andromedavirus*. However, while Anath shared synteny with these phages, several differences were also revealed that suggested a more distant phylogenomic relation, with phage vB_BpsS-36 as its closest known relative. Based on the relatively conserved large terminase gene in the genomes (Fig. 3B), a maximum-likelihood phylogenetic tree was constructed using the MUSCLE algorithm^18^ (default settings) in MEGA7^19^ (Tamura-3 model^20^ (T92) and gamma distributed (+ G)). It included Anath, the entire *Andromedavirus* genus, vB_BpsS-36 and eight other outlying *Bacillus* phages (Fig. 3C). This placed Anath in a separate clade that included vB_BpsS-36 as the only other member. Thus, in agreement with the current guidelines of the International Committee on Taxonomy of Viruses (ICTV) for genus-level classification^21^ (< 50% nucleotide similarity), and considering the differences in nucleotide and protein similarity between Anath and vB_BpsS-36, it is proposed that phage Anath represents a separate and novel genus of *Bacillus* phages.

**Figure 4:**
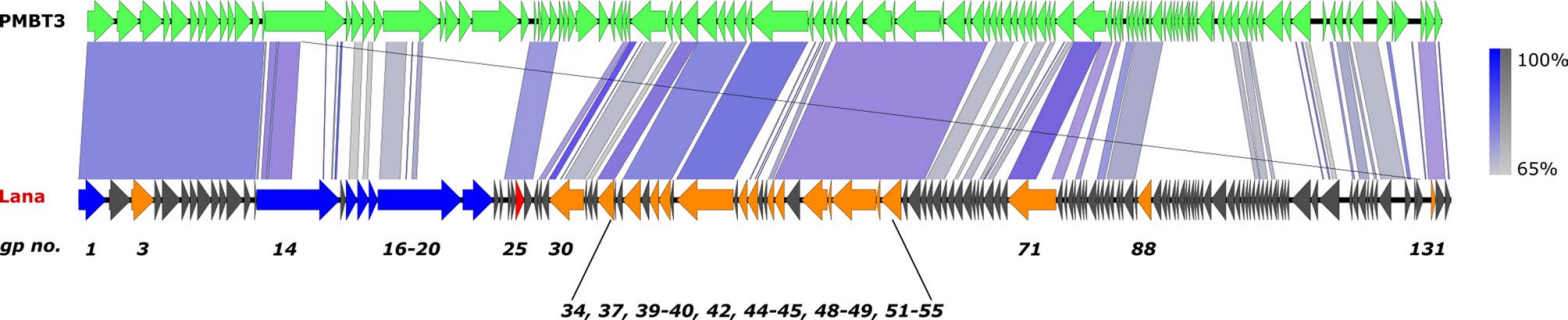
Genome comparison of Lana (bottom) and PMBT3 using the Easyfig genome visualisation tool in BLASTn mode (version 2.2.3). Nucleotide similarity at different regions between genomes is given in percentages (right bar).

For Lana, a Genbank BLASTn search revealed only one single unclassified *Siphoviridae*, *Pseudomonas* phage PMBT3 (accession no. MG596799.1), that shares sequence similarity (57% coverage, 78% nucleotide identity). Sequence alignment between Lana and PMBT3 in BLASTn, standardised to the Lana genome, produced a 44% overall nucleotide similarity, placing Lana in a distinct *Pseudomonas* phage genus^21^. In support of this, a comparison between the Lana and PMBT3 revealed some discrepancies in gene synteny (Fig. 4). Due to the lack of other BLASTn phage hits, no further phylogenomic analyses were undertaken for phage Lana.

To assess the phylogeny of both phages including more distantly related phages and prophages, trees were built based on the conserved nucleotide sequences of the major capsid protein (Anath) and terminase (Anath and Lana). For the analyses phages from a combination of both BLASTp and BLASTn searches were included. However, the results did not provide any new information with regards to the classification. The trees are shown in Figures S6, S7 (Anath) and Figures S8 (Lana) in the Supplementary Information.

### Verification of phage virion proteins

The presence and identity of virion proteins from both phages, harvested from enrichment cultures, were successfully verified. Eight virion proteins were identified for phage Anath: gp2 (portal protein), gp7 (hypothetical protein), gp8 (major capsid protein), gp10 (putative tail fibre), gp15 (hypothetical protein), gp18 (hypothetical protein), gp19 (hypothetical protein) and gp20 (hypothetical protein); and five for phage Lana: gp4 (hypothetical protein), gp5 (hypothetical protein), gp11 (hypothetical protein), gp14 (phage tail protein), and gp19 (phage tail fibre protein). Supplementary Table S4 shows all 136 identified proteins associated with both the host and the phage.

## Conclusions

The sampling method in the present study followed the method applied in the recent study by Korbel et al. (2017)^22^ to investigate the microbiology of groundwater. It recommends that groundwater wells are purged of (at least) three times their volume before a sample is designated as “aquifer” water. Since the wells were purged a minimum of 13 times of their volume before sampling, there can be confidence that bacteriophages indigenous to the reservoir were isolated. In summary, this study revealed for the first time the complete genomes of two novel phages isolated from groundwater, which are likely to represent their own separate genera. Thus, in view of (i) the distant relationship between the Anath and Lana hosts (*Bacillus* and *Pseudomonas,* respectively) and (ii) the present evidence suggesting that the phages represent novel genera, it can be argued that the limited scope of this study further confirms that groundwater reservoirs are an underexplored, heterogenic and poorly understood biotope. This is in line with recent findings that describe aquifers as (i) harbouring diverse microbial communities^1,6,23^ and (ii) acting as viral reservoirs^7^. This also emphasises the great potential of groundwater reservoirs for elucidating novel food web interactions and novel phage discoveries. Therefore, continued effort should be made to further isolate and catalogue phages from subsurface environments in order to contribute significantly to studies of predator–prey interactions and biogeochemical cycling, for example, and to the development of groundwater bioremediation strategies.

## Materials and methods

### Media and culture conditions

All bacterial culturing steps, with or without phages, were undertaken at room temperature (~ 22 °C) using R2A agar and R2B broth (Alpha Biosciences Inc., Baltimore, Maryland, USA) as growth media. Liquid cultures were grown with agitation at 200 rpm. Between uses, bacterial isolates were stored on R2A agar at 4 °C or as cryostocks at − 80 °C (~ 20% glycerol). Purified phage suspensions were stored at 4 °C between uses. The buffer solution (SM buffer) for phage resuspension contained 100 mM NaCl, 8 mM MgSO4 and 50 mM Tris–Cl (pH 7.5) in autoclaved Milli-Q water.

### Bacterial isolation from sampled groundwater

Groundwater from 11 wells at the Skelstofte test sites (Vedby, Denmark, 54°52′34.1ʺN 11°16′18.7ʺE) was sampled at depths of between 7 and 12 m below the surface. These test sites are used to study bioremediation and monitoring strategies of groundwater contamination^24^. Briefly, to obtain water samples representative of the reservoir, wells were purged of ~ 13–22 times their volume (well ø = 4 mm) using a peristaltic pump with a 0.2 L min^−1^ flow rate to avoid disruption of the natural water movement, and stable values were observed for O_2_, pH, conductivity, temperature and other redox parameters before sampling (individual parameters varied between wells) (data not shown). Subsequently, 1-L samples were extracted into sterile glass bottles and stored at 4 °C until use. From the samples, heterotrophic bacteria were isolated on R2A agar (Alpha Biosciences Inc., Baltimore, Maryland, USA), which gave a collection of 84 isolates representing diverse colony morphotypes.

In brief, sample bottles with groundwater were shaken thoroughly and 100 µL of each well sample was plated and left to grow for 4–14 days. Colony growth from samples varied greatly and library building was based on acquiring all the colony morphotypes throughout the incubation period. Where possible, several colonies of a distinct morphotype (up to a maximum of eight) were picked. To obtain pure isolates, colonies were re-streaked three times.

### Phage screening, isolation and amplification

Phage activity was detected in a pre-screening of phage enrichment cultures containing sample water, concentrated media and the relevant isolate. Screening hits, indicated by clear or turbid zones in the top agar layer as a result of plausible phage activity, were then followed by a new enrichment to verify phage activity and subsequently obtain pure phage isolates.

The initial enrichment and pre-screening were performed by mixing 2.5 mL 2 × R2B, 2.5 mL sample water and then inoculating with 50 µL culture of the relevant isolate (isolated from that sample water). Following five days of growth, enrichment cultures were centrifuged at 10,000 *g* for three minutes and supernatant was filtered through a 0.22-µm sterile syringe PVDF membrane filter (Millex Durapore, Burlington, MA, USA). Phage activity in the filtrate from each enrichment was screened in a standard double agar overlay assay. Briefly, 10 µL of each filtered enrichment was drop-plated in 10 replicates onto a semisolid agar, R2B + 50 mM CaCl_2_/MgCl_2_ + 0.6% agarose, inoculated with 100 µL culture of relevant bacterial isolate, using R2A as the bottom agar layer. Plates were incubated at room temperature and inspected daily for one week, with any potential plaque forming noted as ‘hits’ for groundwater sample and isolate.

The follow-up enrichment cultures of phage-host ‘hits’ contained 4 mL 10 × R2B broth, 35 mL groundwater sample, 1 mL culture of relevant host and 10 mM CaCl_2_/MgCl_2_. Enrichment cultures were incubated for 24 h. Subsequently, the culture was supplemented with 1 M NaCl and incubated for 30 min. The culture was then centrifuged at 5,000 g for five minutes and the supernatant was filtered through a 0.45-µm PVDF syringe filter (Millex Durapore, Burlington, MA, USA). Then 100 µL of the filtered supernatant was used in a double agar overlay assay as described above, and individual plaques were picked and re-plated three times to obtain pure phage samples.

A high titre of both phages was obtained by polyethylene glycol (PEG) precipitation as described in^9^ with modifications. Briefly, 200 mL R2B was inoculated with 100 µL host culture, infected with 100 µL of pure phage lysate and left overnight. The following day, 1 M NaCl was added to cultures and left on an orbital shaker for one hour to burst bacterial cells. Cultures were then spun at 12,000 *g* for 10 min, supernatant was collected and PEG was added to reach 10% w/v. The mixture was left for two hours on an orbital shaker for phage-PEG adsorption. Finally, the mixture was centrifuged at 12,000 *g* for 10 min and the pellets with phage virions were resuspended and collected in 3–5 mL SM buffer. Following PEG precipitation, titre was determined by PFU counts by drop-plating dilution series of collected particles on an agar overlayer, as described above. A titre range between 10^10^–10^11^ PFU mL^−1^ was obtained. Single plaques or high-titre phage samples (10^10^–10^11^ PFU mL^−1^) were used in downstream analysis to characterise and determine the phage’s (i) morphology, (ii) burst size, (iii) general genome features (iv) homology with other related phages, and (v) virion proteins. For TEM imaging and peptide sequencing, phage samples were further purified by a caesium chloride gradient according to the protocol of Clokie & Kropinski^25^.

### Host DNA extraction, sequencing and identification

DNA was extracted from 5 mL of each host culture (grown for five days) using an Ultraclean Microbial DNA Isolation Kit (Mo bio Laboratories, Carlsbad, California, USA), following the manufacturer’s protocol. DNA libraries were prepared with the Nextera XT DNA kit (Illumina, San Diego, USA) according to the manufacturer’s protocol. The prepared libraries were then sequenced in a 2 × 251 paired-end sequencing run, as part of a flow cell, using the Illumina MiSeq v2 kit (Illumina, San Diego, CA, USA). In assembling host draft genomes, CutAdapt (v1.8.3) was used to quality-trim sequence reads (bases with < q20 removed from read ends) and to remove any contaminants (primers and indexes). Shorter reads (< 50 bp) were then removed and overlapping read pairs merged using AdapterRemoval (v2.1.0)^26^ at default settings. Finally, the cleaned merged and unmerged reads were assembled using SPAdes (v3.6.0)^27^ and assemblies evaluated in QUAST (v3.1)^28^.

For each host, the genome sequence data were uploaded to the Type (Strain) Genome Server (TYGS), a free bioinformatics platform available at https://tygs.dsmz.de, for a whole genome-based taxonomic analysis^8^. Methods (and results) for strain identification are provided in the Supplementary Information.

### Transmission electron microscopy of virions

The caesium chloride-purified phage sample was adsorbed to freshly prepared ultra-thin carbon film and fixed with 2% (v/v) EM-grade glutaraldehyde (20 min). Fixed samples were then negatively stained with 1% (w/v) uranyl acetate and picked up with 400-mesh copper grids (Plano, Wetzlar, Germany). Finally, prepared samples were analysed using a Tecnai 10 transmission electron microscope (Thermo Fisher, Eindhoven, the Netherlands) at an acceleration voltage of 80 kV. Micrographs were taken with a MegaView G2 CCD-camera (EMSIS, Muenster, Germany).

### Phage Lana burst size

Phage latency period and burst size were determined for phage Lana in a one-step growth curve experiment as described elsewhere^9^. Here, the host was grown to OD_600nm_ 0.75, corresponding to 4 × 10^7^ CFUs mL^−1^. Then, 10 mL culture was infected with a 0.05 multiplicity of infection and incubated for 20 min at 200 rpm to allow phage-host adsorption. After adsorption, three aliquots of infected culture were diluted × 10,000, and PFUs in the three diluted cultures were followed over time to determine the phage latency period and phage burst size. Experimental cultures were sampled at the beginning of the experiment and then every 10 min from 90 min until the end. Average PFU numbers before and after the burst event were used to calculate the burst size. In calculating the burst size, the infection efficacy of phage Lana after the adsorption step was also considered. After adsorption, one sample from the infected culture was centrifuged at 6,000 *g* for five minutes, thereby separating infecting phages (pellet) and unadsorped phages (supernatant). Subsequently, three technical replicates from the resuspended pellet and the supernatant were plated and PFUs were counted. The infection efficacy of Lana was then determined to be 19.27% ± 1.04 SEM. Prior to the experiment, the study established (i) a growth curve for the host strain to determine the OD_600nm_—CFU relationship and (ii) an approximate phage latency period (data not shown).

### Phage DNA extraction and genome sequencing

For both phages the protocol for the direct plaque sequencing (DPS) method was used as described by Kot et al.^29^ with the following modifications for phage Anath only: 500 µL high titre lysate was used as input (~ 2 × 10^10^ PFU mL^−1^), 10 µL (100 mg mL^−1^) of protein kinase K (Thermo Scientific, Waltham, USA) was used in capsid DNA release, and 10 µL was used as elution volume for purified DNA. Phage DNA libraries were prepared with the Nextera XT kit DNA kit (Illumina, San Diego, USA), using the DPS method described in Kot et al.^29^ for phage Lana and the manufacturer’s kit protocol for phage Anath. Prepared libraries were sequenced in a 2 × 250 paired-end sequencing run, as part of a flow cell, using the Illumina MiSeq platform (Illumina, San Diego, USA).

### Phage genome assembly and annotation

Sequence reads were trimmed and assembled in the CLC Genomic Workbench 11.1.0 (CLC bio, Aarhus, Denmark) using standard settings, and assembly was cross-verified using SPAdes (version 3.13.0, using trimmed and merged reads as input running on-careful mode), as described elsewhere^30^. Assembled genomes were automatically annotated using the RAST online tool^31^ and were manually curated by cross-referencing with four other publicly available protein recognition tools: BLASTp, Pfam, HHpred and Phyre^32–35^. Predicted protein functions were annotated accordingly when identical functions were predicted in at least three of the five databases used.

### De novo peptide sequencing—identification of structural proteins

To identify the proteins, the previous procedure for protein purification from Lavigne et al.^36^ was followed with minor modifications. In short, 100 µL of the phage extract was transferred to an Amicon Ultra filter unit (MWCO 30 kDa) and centrifuged at 14,000 × *g* for 20 min and further desalted four times with 450 µL water. The filtrate containing the phage particles (10 µL) was denaturised in 25 µL buffer consisting of 6 M urea, 5 mM dithiothreitol and 50 mM Tris–HCl (pH 8). The phage particles were destabilised by five successive freeze-thawing cycles followed by a full hour incubation at 60 °C to reduce the phage proteins. The proteins were alkylated by adding 25 µL of 100 mM iodoacetamide and 150 µL of 50 mM ammonium bicarbonate and incubated for 45 min at room temperature. Phage proteins were digested with 0.8 µg trypsin dissolved in 40 µL 50 mM ammonia bicarbonate and incubated for 24 h at 37 °C. The protein digest was diluted with 200 µL 0.1% trifluoroacetic acid (TFA) and purified by solid-phase extraction using 2 mg hydrophobic reversed phase well-plate cartridges (Thermo Fisher Scientific) preconditioned with 200 µL acetonitrile and 200 µL 0.1% TFA. The peptides were eluted from the cartridges with two times 25 µL 70% acetonitrile and diluted with 150 µL 0.1% TFA. The phage peptides were analysed using an Ultimate 3,000 RSLCnano UHPLC system hyphenated with a Q Exactive HF mass spectrometer (Thermo Fisher Scientific, Denmark). An amount of 6.4 µL of the sample was loaded on a preconcentration trap (C_18_ 300 µm × 5 mm cartridge, Thermo Fisher Scientific) and eluted onto an analytical column (75 µm × 250 mm, 2 µm C_18_, Thermo Fisher Scientific) with a chromatographic triple-phasic 53 min gradient ranging from 1 to 64% mobile phase B (98% acetonitrile and 0.1% formic acid) at a 300 nL per minute flow rate. The total analysis time was 65 min and mobile phase A consisted of 2% acetonitrile and 0.1% formic acid. The high-resolution mass spectrometer was operated with positive electrospray ionisation in data-dependent mode by automatically switching between MS and MS/MS fragmentation. Based on a survey MS scan in the Orbitrap operated at a mass resolution of 120,000 at m/z 200 with a target of 3e6 ions and a maximum injection time at 50 ms, the twelve most intense peptide ions were selected for MS/MS fragmentation in subsequent scans. The selected ions were isolated (in a m/z 1.4 window) and higher-energy collision dissociation was performed at a normalised collision energy (28) and fragments recorded in centroid mode at a resolution of 60,000 (m/z 200) with a 250 ms maximum filling time and target of 1e5 ions. The high-resolution data generated were analysed in Proteome Discoverer 2.2 (Thermo Fisher Scientific) and searched against predicted phage/host proteins by the Sequest HT algorithm in an iterative processing pipeline. The search criteria were enzyme, trypsin (full); dynamic modifications, methionine oxidation and acetyl (N-terminus); precursor mass tolerance, 5 ppm; fragment mass tolerance, 20 mDa. The processed data were filtered in a Proteome Discoverer consensus workflow with the Peptide Validator algorithm (q-value < 0.01) to ensure the peptide-spectrum match had a false discovery rate under 1%. The de novo peptide sequencing identified 136 proteins in total with a false discovery rate < 1%. Individual samples contained proteins mapping to predicted proteins of both the phage and its host: 17 identified proteins in Anath/*B. mycoides* and 122 identified proteins in Lana/*Pseudomonas* sp. To avoid false positive identification, only phage proteins identified with quality scores (sequest HT score) exceeding the highest quality score of identified *host* proteins were regarded as virion proteins.

## Supplementary information

Supplementary information
