# Supplementary material for: Two novel bacteriophage genera from a groundwater reservoir highlight subsurface environments as underexplored biotopes in bacteriophage ecology
Source: Sci Rep. 2020 Jul 17;10:11879. doi: 10.1038/s41598-020-68389-1 (PMC7368026; doi:10.1038/s41598-020-68389-1)
Supplement: Supplementary file 1 — Supplementary information [file 41598_2020_68389_MOESM1_ESM.docx]

**Supplementary Information - Supporting Results**

**Title:**

*Two novel bacteriophage genera from a groundwater reservoir highlight subsurface environments as underexplored biotopes in bacteriophage ecology*

**Authors:**

Ole Hylling^1^, Alexander B. Carstens^1,2^, Witold Kot^1,2^, Martin Hansen^1^, Horst Neve^3^, Charles M.A.P Franz^3^, Anders Johansen^1^, Lea Ellegaard-Jensen^1^ & Lars H. Hansen^1,2^*

**Affiliation:**

^1^ Aarhus University, Dept. of Environmental Science, Frederiksborgvej 399, 4000 Roskilde, Denmark

^2^ University of Copenhagen, Dept. of Plant- and Environmental Sciences, Section for Microbial Ecology and Biotechnology, Copenhagen, Denmark

^3^ Max Rubner-Institut, Dept. of Microbiology and Biotechnology, Hermann-Weigmann-Straße 1, 24103 Kiel, Germany

^*^**Corresponding author:**

Lars Hestbjerg Hansen ([lhha@plen.ku.dk](mailto:lhha@plen.ku.dk)), University of Copenhagen, Department of Plant- and Environmental Sciences, Thorvaldsensvej 40, 1871 Frederiksberg, Denmark

**Host strain identification**

**Methods and results**

In brief, the TYGS analysis^1^ (March 2020) used to determine the closest type strain genomes was performed in two complementary ways. First, each genome were compared with all type strain genomes available in the TYGS database via the MASH algorithm, a fast approximation of intergenomic relatedness^2^, and ten type strains with the smallest MASH distances were chosen per host genome. Second, an additional set of ten closely related type strains was determined via 16S rDNA gene sequences. These were extracted from the host genomes using RNAmmer^3^ and each sequence was subsequently BLASTed^4^ against the 16S rDNA gene sequence of each of the 11236 type strains currently available in the TYGS database. This was used as a proxy to find the best 50 matching type strains (according to the bitscore) for each host genome and to subsequently calculate precise distances using the Genome BLAST Distance Phylogeny approach (GBDP) under the algorithm ‘coverage’ and distance formula *d_5_*^5^. These distances were finally used to determine the 10 closest type strain genomes for each of the host genome.

The resulting intergenomic distances were used to infer a balanced minimum evolution tree with branch support via FASTME 2.1.4^6^ including SPR postprocessing. Branch support was inferred from 100 pseudo-bootstrap replicates each. The trees were rooted at the midpoint^7^ and visualised with PhyD3^8^**.**


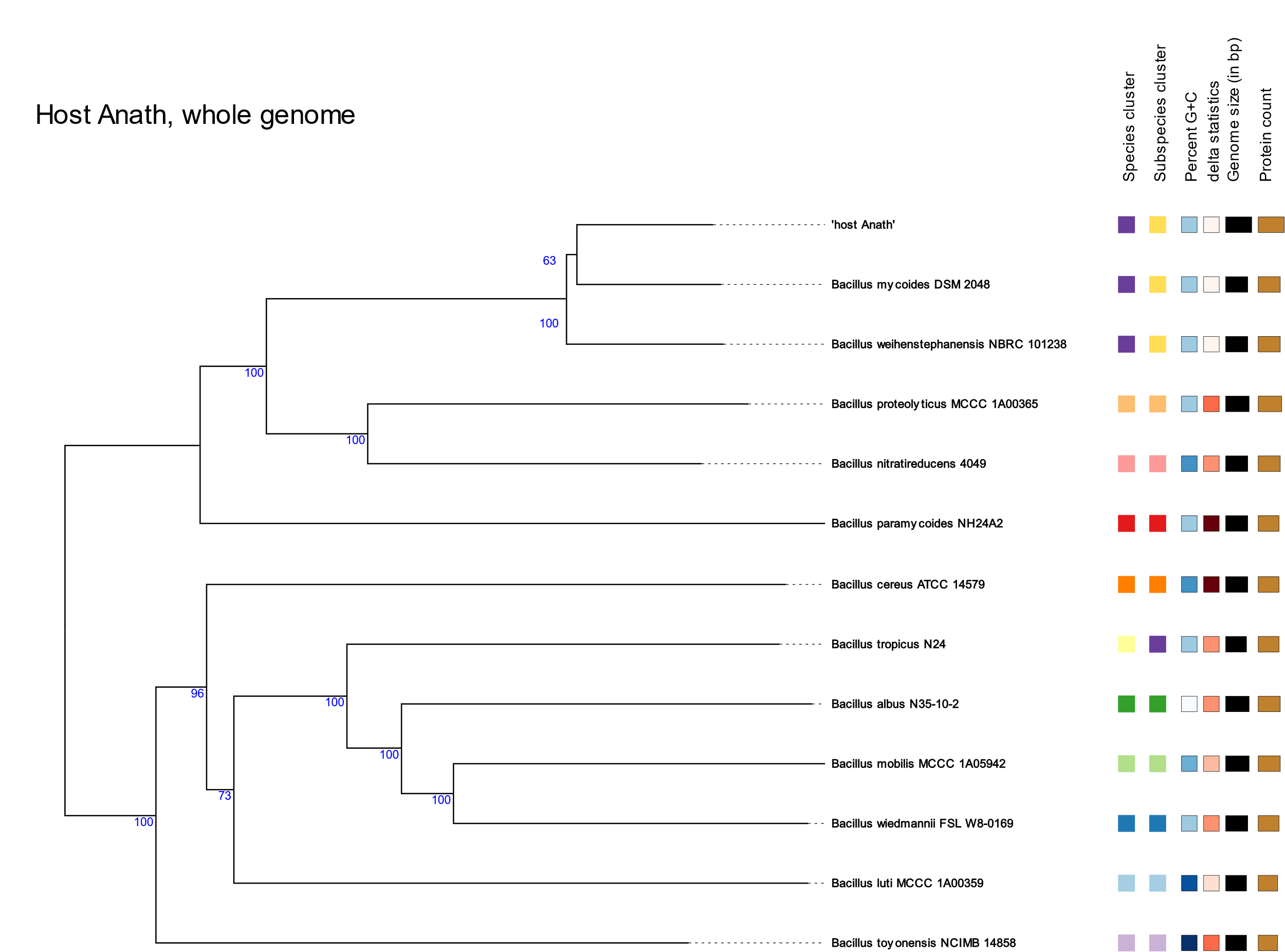


**Figure S1.** Whole genome phylogenetic tree of host Anath and its closest relatives in TYGS^1^. Tree inferred with FastME 2.1.6.1^6^ from GBDP distances calculated from genome sequences. The branch lengths are scaled in terms of GBDP distance formula *d_5_*. The numbers above branches are GBDP pseudo-bootstrap support values > 60 % from 100 replications, with an average branch support of 93.2 %. The tree was rooted at the midpoint^7^.


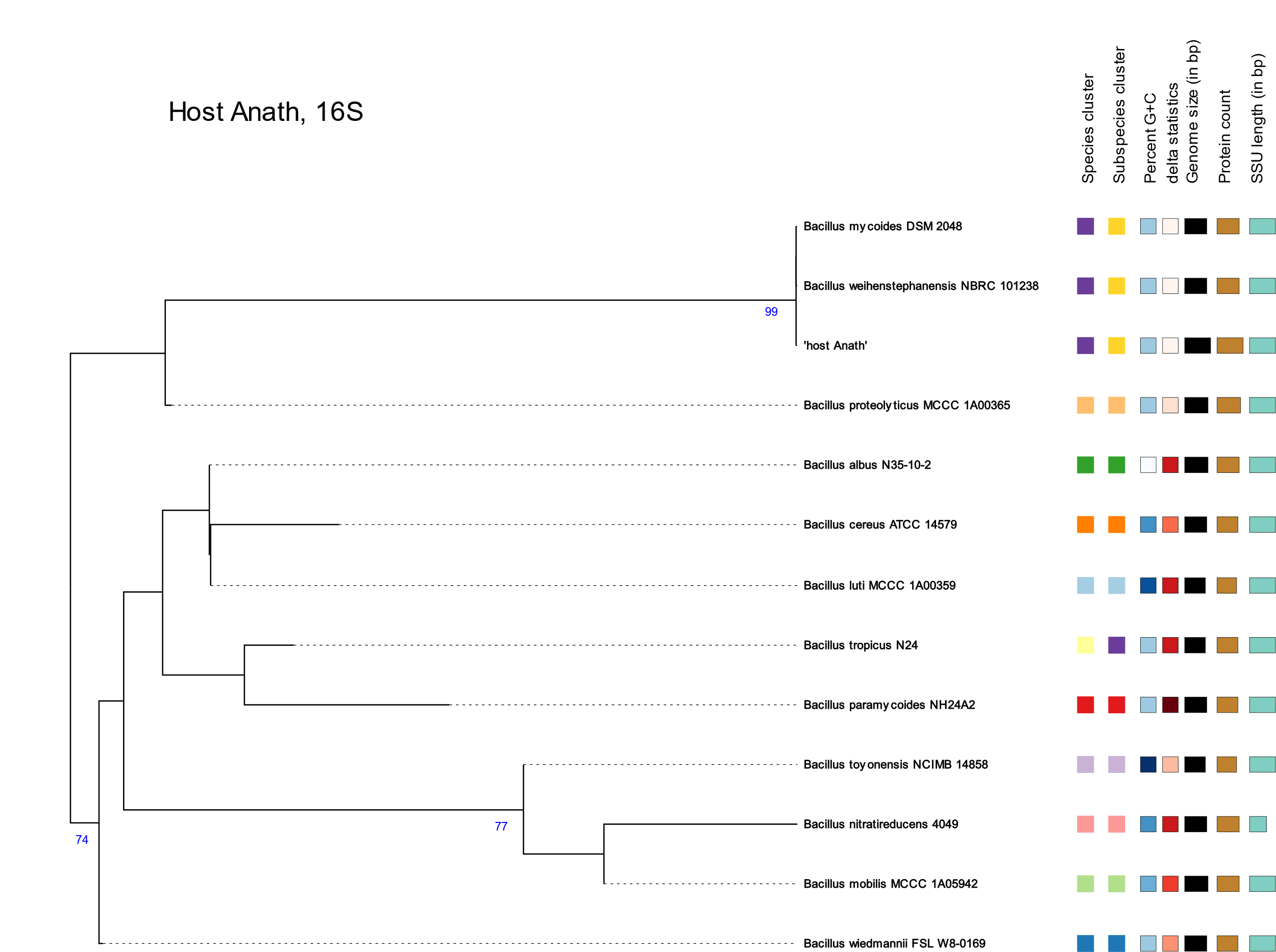


**Figure S2.** 16S phylogenetic tree of host Anath and its closest relatives in TYGS^1^. Tree inferred with FastME 2.1.6.1^6^ from GBDP distances calculated from 16S rDNA gene sequences. The branch lengths are scaled in terms of GBDP distance formula *d_5_*. The numbers above branches are GBDP pseudo-bootstrap support values > 60 % from 100 replications, with an average branch support of 45.1 %. The tree was rooted at the midpoint^7^


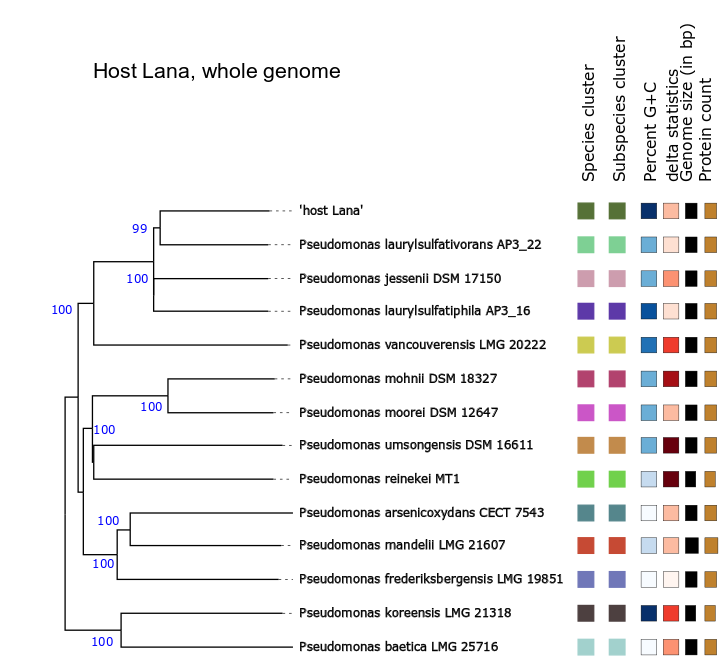
**Figure S3.** Whole genome phylogenetic tree of host Lana and its closest relatives in TYGS^1^. Tree inferred with FastME 2.1.6.1^6^ from GBDP distances calculated from genome sequences. The branch lengths are scaled in terms of GBDP distance formula *d_5_*. The numbers above branches are GBDP pseudo-bootstrap support values > 60 % from 100 replications, with an average branch support of 84.9 %. The tree was rooted at the midpoint^7^.


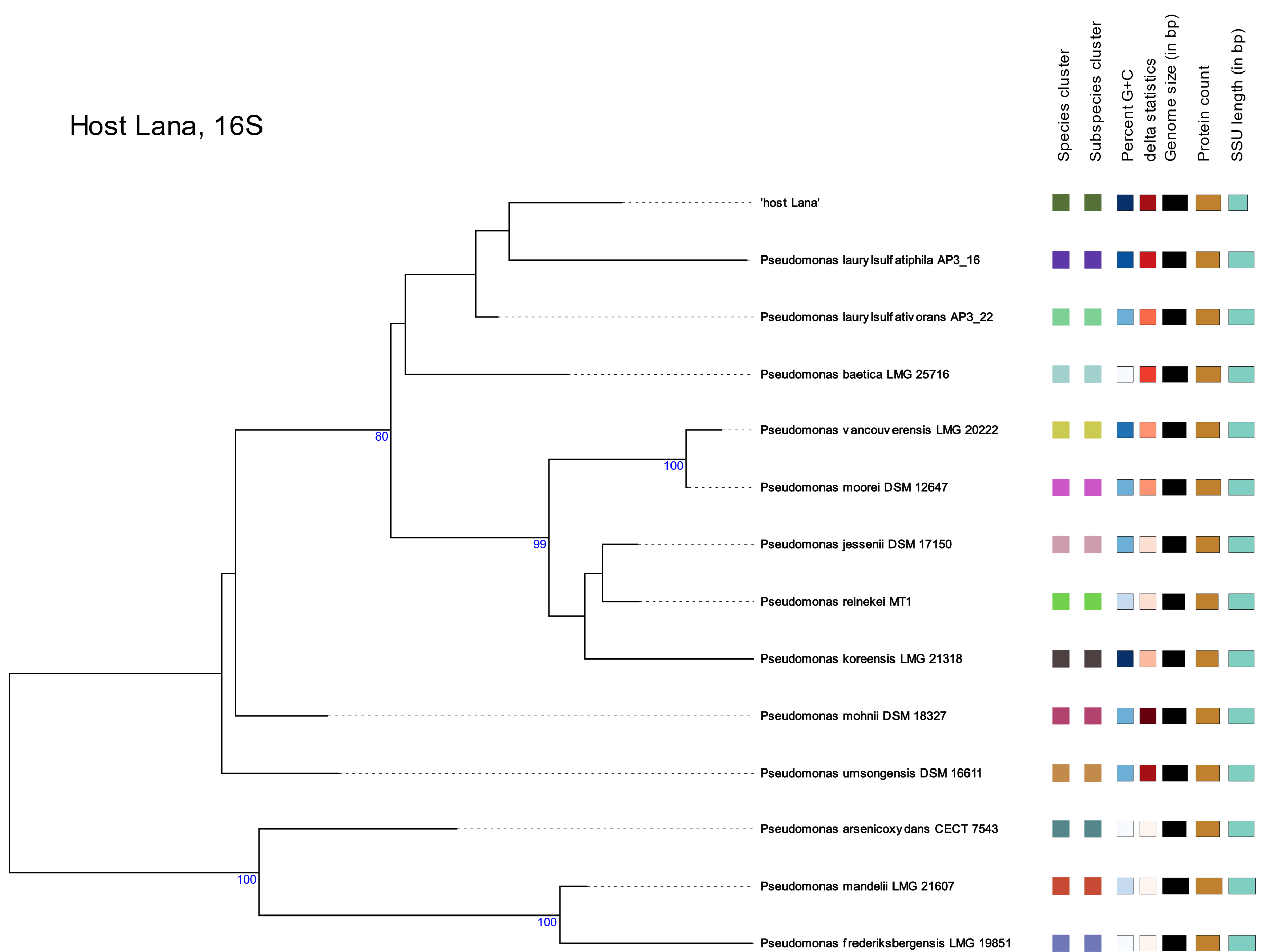


**Figure S4.** 16S phylogenetic tree of host Lana and its closest relatives in TYGS^1^. Tree inferred with FastME 2.1.6.1^6^ from GBDP distances calculated from 16S rDNA gene sequences. The branch lengths are scaled in terms of GBDP distance formula d5. The numbers above branches are GBDP pseudo-bootstrap support values > 60 % from 100 replications, with an average branch support of 66.3 %. The tree was rooted at the midpoint^7^.


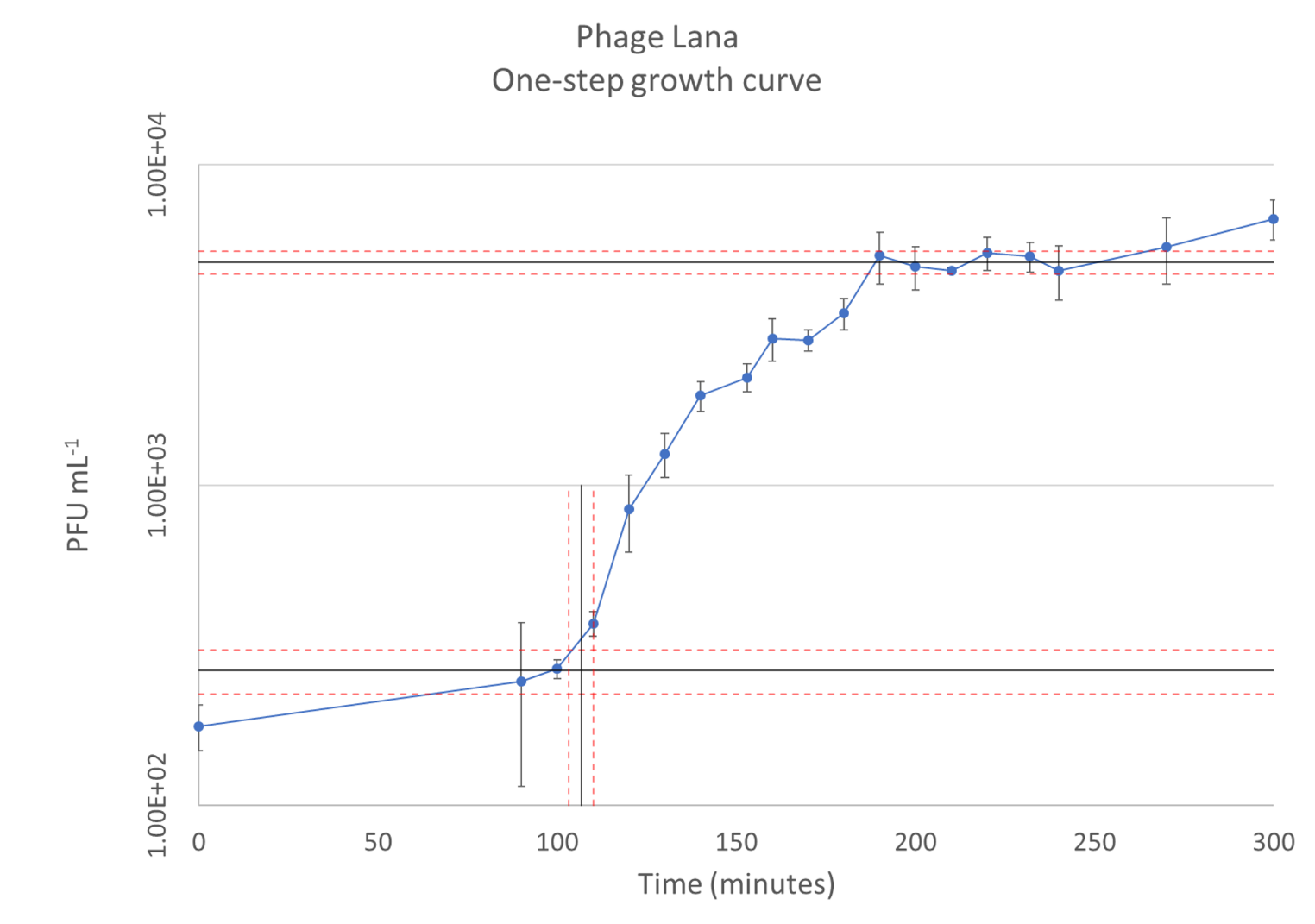


**Figure S5.** Log-plot of the one-step growth curve of the Lana phage, showing progression of PFU mL^-1^ over time (blue plot) with error bars showing standard error of the mean (SEM). Average PFUs mL^-1^ *before* and *after* burst are shown as horizontal solid black lines, with their SEM values as dashed red lines. The latency period was manually evaluated by inspection of data and plotted as a vertical solid line, with SEM as dashed red lines. The time period 0-110 mins was defined as *before* the phage burst, and 190-240 mins was defined as *after* the first phage burst event.

| **Table S1** – Overview of *Bacillus mycoides* phage Anath gene products and their predicted functions. Gene functions predicted as “hypothetical proteins” are not shown. | | | |
| --- | --- | --- | --- |
| Gene product no. | Product length (bp) | Strand | Predicted protein function |
| *gp1* | 1682 | *+* | phage terminase, large subunit |
| *gp2* | 1334 | *+* | portal protein |
| *gp5* | 785 | *-* | antirepressor protein |
| *gp8* | 860 | *+* | major capsid protein |
| *gp10* | 1823 | *+* | phage protein (DUF2479) putative tail fibre |
| *gp23* | 362 | *+* | N-acetylmuramoyl-L-alanine amidase |
| *gp29* | 1289 | *+* | replicative DNA helicase |
| *gp32* | 1013 | *+* | DNA primase |
| *gp34* | 2315 | *+* | ribonucleoside-diphosphate reductase sub-unit alpha |
| *gp35* | 1040 | *+* | ribonucleoside-diphosphate reductase sub-unit beta |
| *gp38* | 3188 | *+* | DNA polymerase III alpha subunit |
| *gp40* | 767 | *+* | PD-(D/E)XK nuclease superfamily |
| *gp41* | 608 | *+* | restriction endonuclease-like |
| *gp42* | 707 | *+* | RNA polymerase sigma factor |
| *gp48* | 737 | *+* | thymidylate synthase ThyX |
| *gp54* | 578 | *+* | nucleotide/nucleoside kinase |
| *gp55* | 458 | *+* | pyrophosphatase (MazG-like) |
| *gp60* | 1250 | *+* | cell division protein FtsK |
| *gp73* | 1280 | *+* | protein kinase (protein linked receptor kinase) |

***Annotated genes: Bacillus mycoides* phage Anath**

| **Table S2** – Overview of *Pseudomonas* sp. phage Lana gene products and their predicted functions. Gene functions predicted as “hypothetical proteins” are not shown. | | | |
| --- | --- | --- | --- |
| Gene product no. | Product length (bp) | Strand | Predicted protein function |
| *gp1* | 1787 | *+* | phage terminase, large subunit |
| *gp3* | 1487 | *-* | peptidase protein |
| *gp14* | 5369 | *-* | phage tail protein |
| *gp16* | 725 | *-* | phage minor tail protein |
| *gp17* | 737 | *-* | phage tail assembly protein |
| *gp18* | 581 | *-* | phage tail assembly protein |
| *gp19* | 5414 | *-* | phage tail fibre protein |
| *gp20* | 2000 | *-* | phage tail fibre protein |
| *gp25* | 542 | *-* | lysozyme |
| *gp30* | 2267 | *-* | DNA binding protein |
| *gp34* | 1073 | *-* | exonuclease |
| *gp37* | 1148 | *-* | phage DNA ligase |
| *gp39* | 602 | *-* | nucleoside kinase |
| *gp40* | 746 | *-* | 3'-5' Exonuclease |
| *gp42* | 3665 | *-* | DNA polymerase l |
| *gp44* | 587 | *-* | 3'-5' exoribonuclease |
| *gp45* | 695 | *-* | thymidylate synthase |
| *gp48* | 440 | *-* | TerB-like protein |
| *gp49* | 620 | *+* | MazG-like protein |
| *gp51* | 1652 | *+* | helicase |
| *gp52* | 227 | *+* | putative RNA polymerase |
| *gp53* | 2888 | *+* | putative primase-polymerase protein |
| *gp54* | 248 | *+* | putative RNA transcription factor |
| *gp55* | 1328 | *+* | DNA helicase |
| *gp71* | 3143 | *+* | DNA modification methylase |
| *gp88* | 914 | *+* | transporter-like phage protein |
| *gp131* | 290 | *+* | DNA binding protein |

***Annotated genes: Pseudomonas* sp. phage Lana**

|  |  |  |  |  |  |  |  |
| --- | --- | --- | --- | --- | --- | --- | --- |
| **Table S3** – Overview of best hits with phage Anath in a BLASTn search | | | | | | | |
| **Name** | **Genus** | **Host** | **Source** | **% cov.** | **% ident.** | **E-value** | **Accession** |
| vB_BpsS-36 | *Unclassified* | *B. pseudalcaliphilus* | Lake | 22 | 70.02 | 0.0 | MH884513.1 |
| Curly | *Andromedavirus* | *B. pumilus* | Soil | 15 | 70.10 | 2e-121 | KC330679.1 |
| Andromeda | *Andromedavirus* | *B. pumilus* | Soil | 14 | 70.22 | 2e-121 | KC330684.1 |
| Gemini | *Andromedavirus* | *B. pumilus* | Soil | 14 | 70.22 | 2e-121 | KC330681.1 |
| Glittering | *Andromedavirus* | *B. pumilus* | na. | 13 | 70.68 | 9e-127 | KF669651.1 |
| Leo2 | *Andromedavirus* | *B. pumilus* | Soil | 13 | 70.36 | 1e-124 | KU836751.1 |
| Finn | *Andromedavirus* | *B. pumilus* | Soil | 13 | 70.22 | 1e-118 | KC330683.1 |
| Taylor | *Andromedavirus* | *B. pumilus* | Soil | 13 | 71.20 | 9e-133 | KC330682.1 |
| Eoghan | *Andromedavirus* | *B. pumilus* | Soil | 13 | 71.20 | 9e-133 | KC330680.1 |
| Riggi | *Andromedavirus* | *B. pumilus* | na. | 12 | 69.85 | 3e-113 | KF669659.1 |
| Blastoid | *Andromedavirus* | *B. pumilus* | na. | 12 | 70.15 | 5e-123 | KF669648.1 |
|  |  |  |  |  |  |  |  |

***Phage Anath: BLASTn results***

***
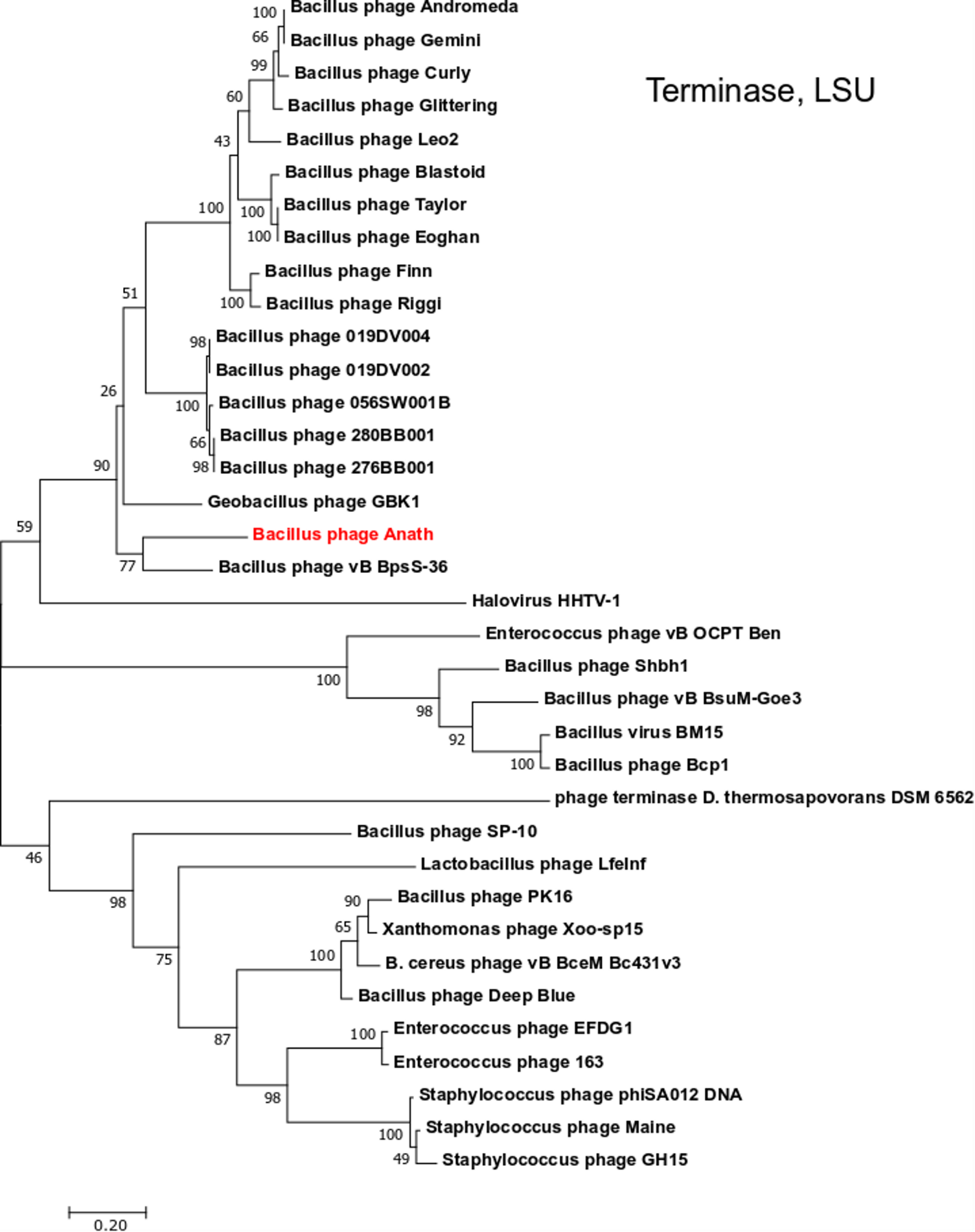
***

**Figure S6**. Molecular phylogenetic analysis by the maximum likelihood method of combined BLASTn and BLASTp phage hits of phage Anath; terminase nucleotide sequences. The evolutionary history was inferred using the maximum likelihood method based on the Tamura 3-parameter model^4^. The tree with the highest log likelihood (-20190.68) is shown. The percentage of trees in which the associated taxa clustered together is shown next to the branches (100 replicates). Initial tree(s) for the heuristic search were obtained automatically by applying neighbour-joining and BioNJ algorithms to a matrix of pairwise distances estimated using the maximum composite likelihood (MCL) approach, and then selecting the topology with superior log likelihood value. A discrete Gamma distribution was used to model evolutionary rate differences between sites (five categories (+G, parameter = 2.4738)). The tree is drawn to scale, with branch lengths measured in the number of substitutions per site. The analysis involved 36 nucleotide sequences. Codon positions included were 1st+2nd+3rd+Noncoding. All positions containing gaps and missing data were eliminated. There were a total of 980 positions in the final dataset. Evolutionary analyses were conducted in MEGA7^5^.

**
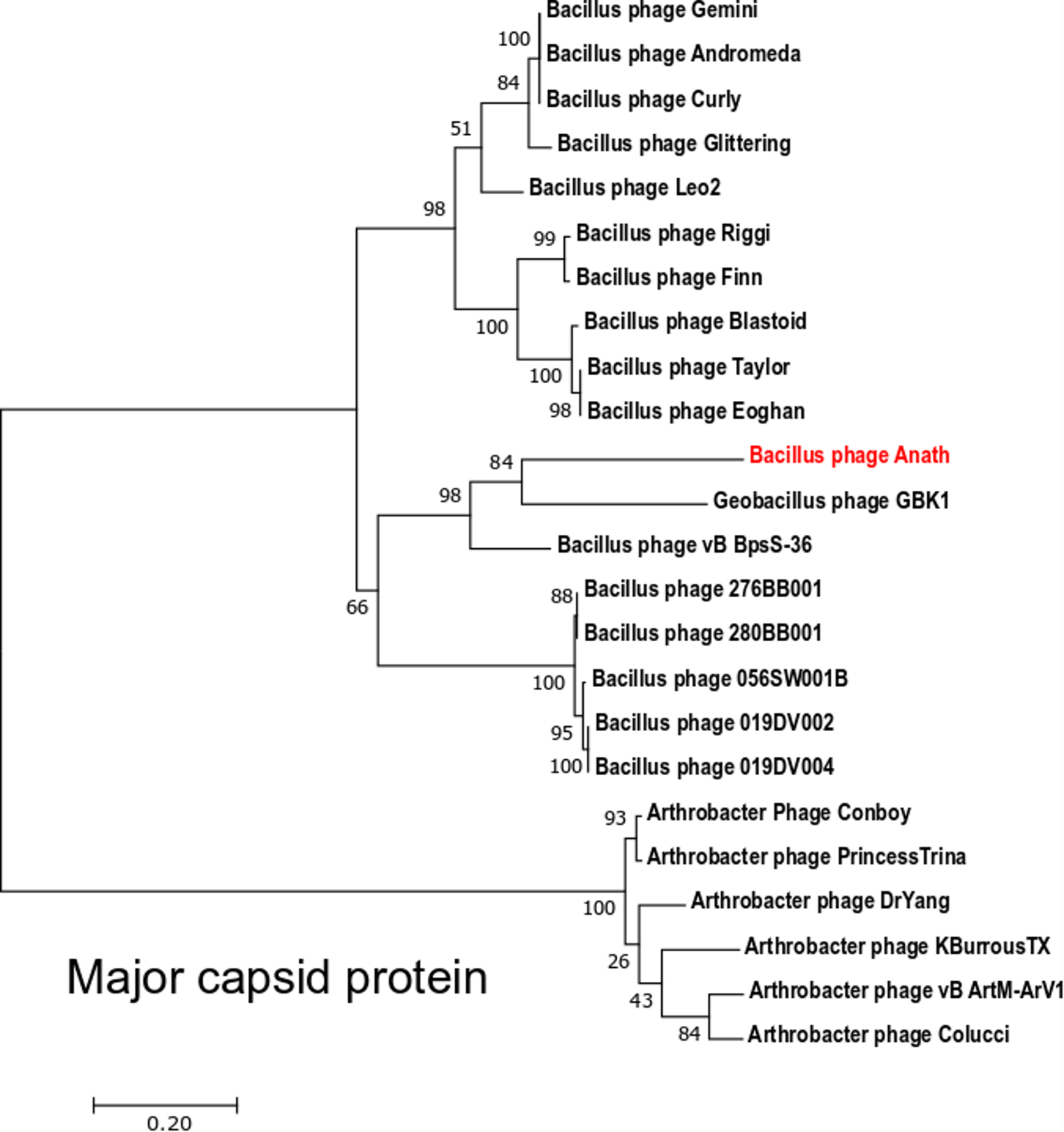
**

**Figure S7**. Molecular phylogenetic analysis by the maximum likelihood method of combined BLASTn and BLASTp phage hits of phage Anath; major capsid (protein) nucleotide sequence. The evolutionary history was inferred by using the maximum likelihood method based on the Kimura 2-parameter model^6^. The tree with the highest log likelihood (-7021.42) is shown. The percentage of trees in which the associated taxa clustered together is shown next to the branches (100 replicates). Initial tree(s) for the heuristic search were obtained automatically by applying neighbour- joining and BioNJ algorithms to a matrix of pairwise distances estimated using the maximum composite likelihood (MCL) approach, and then selecting the topology with superior log likelihood value. A discrete Gamma distribution was used to model evolutionary rate differences between sites (five categories (+G, parameter = 0.9809)). The tree is drawn to scale, with branch lengths measured in the number of substitutions per site. The analysis involved 24 nucleotide sequences. Codon positions included were 1st+2nd+3rd+Noncoding. All positions containing gaps and missing data were eliminated. There were a total of 736 positions in the final dataset. Evolutionary analyses were conducted in MEGA7^5^.

***
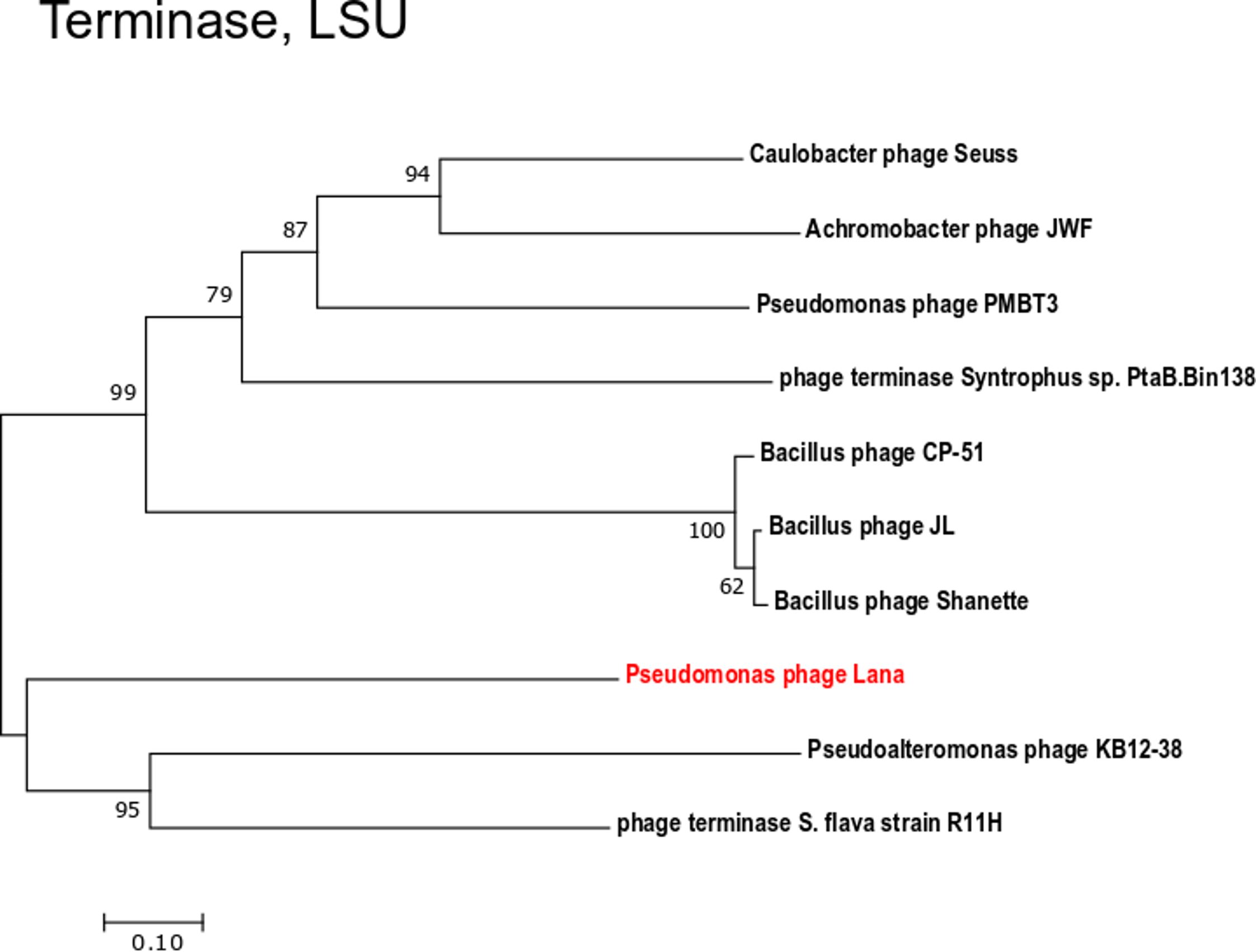
***

**Figure S8**. Molecular phylogenetic analysis by the maximum likelihood method of combined BLASTn and BLASTp phage hits of phage Lana; terminase nucleotide sequences. The evolutionary history was inferred by using the maximum likelihood method based on the Tamura 3-parameter model^4^. The tree with the highest log likelihood (-8577.67) is shown. The percentage of trees in which the associated taxa clustered together is shown next to the branches (100 replicates). Initial tree(s) for the heuristic search were obtained automatically by applying neighbour-joining and BioNJ algorithms to a matrix of pairwise distances estimated using the maximum composite likelihood (MCL) approach, and then selecting the topology with superior log likelihood value. A discrete Gamma distribution was used to model evolutionary rate differences between sites (five categories (+G, parameter = 4.4306)). The tree is drawn to scale, with branch lengths measured in the number of substitutions per site. The analysis involved 10 nucleotide sequences. All positions containing gaps and missing data were eliminated. There were a total of 866 positions in the final dataset. Evolutionary analyses were conducted in MEGA7^5^.

| **Table S4**. Mass spectrometry-based de novo peptide sequencing for identification of proteins in purified phage samples. In protein identification, peptides were mapped to genomic protein FASTAs of phage Anath, phage Lana and their hosts, *B. mycoides* and *Pseudomonas sp*. respectively. Identified phage proteins, highlighted as bold, exhibit Sequest HT scores above the highest scoring host proteins (highlighted as red); protein #34 (*B. mycoides*) in the Anath/*B. mycoides* sample and protein #64 (*Pseudomonas sp*). in the Lana/*Pseudomonas* sp. sample respectively. Proteins predicted as structural phage proteins are marked with a **•** symbol. All listed proteins showed a high confidence in false-discovery rate (<1 %). PSMs = peptide spectrum matches. | | | | | | | | | | |
| --- | --- | --- | --- | --- | --- | --- | --- | --- | --- | --- |
| # of total | # of ref. |  | Reference | Annotation/gene product (gp) | Sequest HT score | Coverage [%] | #Peptides | #PSMs | Abundance | |
|  |  |  |  |  |  |  |  |  | Lana sample | Anath sample |
| 1. | 1. | **•** | phage Anath | **portal protein (gp2)** | **17.2** | **19** | **8** | **13** | **not found** | **high** |
| 2. | 2. |  | phage Anath | hypothetical protein (gp6) | 0 | 7 | 2 | 2 | not found | high |
| 3. | 3. |  | phage Anath | **hypothetical protein (gp7)** | **159.27** | **46** | **6** | **54** | **high** | **high** |
| 4. | 4. | **•** | phage Anath | **major capsid protein (gp8)** | **985.86** | **68** | **21** | **451** | **high** | **high** |
| 5. | 5. | **•** | phage Anath | **putative tail fibre (gp10)** | **7.88** | **7** | **3** | **5** | **not found** | **high** |
| 6. | 6. |  | phage Anath | **hypothetical protein (gp15)** | **49.01** | **28** | **5** | **19** | **high** | **high** |
| 7. | 7. |  | phage Anath | **hypothetical protein (gp18)** | **4.98** | **3** | **3** | **4** | **not found** | **high** |
| 8. | 8. |  | phage Anath | **hypothetical protein (gp19)** | **15.62** | **9** | **6** | **9** | **peak found** | **high** |
| 9. | 9. |  | phage Anath | **hypothetical protein (gp20)** | **7.39** | **5** | **3** | **4** | **not found** | **high** |
| 10. | 1. |  | phage Lana | hypothetical protein (gp2) | 84.97 | 20 | 9 | 33 | high | not found |
| 11. | 2. |  | phage Lana | peptidase protein (gp3) | 24.71 | 9 | 4 | 11 | high | not found |
| 12. | 3. |  | phage Lana | **hypothetical protein (gp4)** | **229.94** | **49** | **6** | **79** | **high** | **peak found** |
| 13. | 4. |  | phage Lana | **hypothetical protein (gp5)** | **1211.33** | **61** | **15** | **397** | **high** | **peak found** |
| 14. | 5. |  | phage Lana | hypothetical protein (gp7) | 22.69 | 30 | 3 | 9 | high | not found |
| 15. | 6. |  | phage Lana | hypothetical protein (gp8) | 32.59 | 35 | 6 | 11 | high | peak found |
| 16. | 7. |  | phage Lana | hypothetical protein (gp9) | 2.03 | 7 | 1 | 1 | high | not found |
| 17. | 8. |  | phage Lana | hypothetical protein (gp10) | 20.88 | 18 | 3 | 7 | high | not found |
| 18. | 9. |  | phage Lana | **hypothetical protein (gp11)** | **590.27** | **64** | **17** | **220** | **high** | **peak found** |
| 19. | 10. | **•** | phage Lana | **phage tail protein (gp14)** | **405.4** | **34** | **50** | **144** | **high** | **peak found** |
| 20. | 11. |  | phage Lana | hypothetical protein (gp15) | 5.71 | 7 | 1 | 2 | peak found | not found |
| 21. | 12. | **•** | phage Lana | phage minor tail protein (gp16) | 18.3 | 22 | 5 | 13 | high | peak found |
| 22. | 13. | **•** | phage Lana | phage tail assembly protein (gp18) | 12.66 | 18 | 3 | 6 | high | not found |
| 23. | 14. | **•** | phage Lana | **phage tail fibre protein (gp19)** | **363.67** | **30** | **36** | **125** | **high** | **peak found** |
| 24. | 15. | **•** | phage Lana | phage tail fibre protein (gp20) | 68.31 | 19 | 8 | 22 | high | peak found |
| 25. | 16. |  | phage Lana | hypothetical protein (gp26) | 15.52 | 28 | 4 | 7 | high | not found |
| 26. | 17. |  | phage Lana | phage DNA ligase (gp37) | 1.99 | 7 | 2 | 2 | high | not found |
| 27. | 18. |  | phage Lana | TerB-like protein (gp48) | 23.7 | 26 | 2 | 8 | high | not found |
| 28. | 19. |  | phage Lana | putative primase-polymerase protein (gp53) | 1.68 | 2 | 1 | 1 | high | not found |
| 29. | 20. |  | phage Lana | transporter-like phage protein (gp88) | 13.19 | 41 | 7 | 8 | high | peak found |
| 30. | 21. |  | phage Lana | hypothetical protein (gp120) | 2.14 | 2 | 1 | 1 | high | not found |
| 31. | 22. |  | phage Lana | hypothetical protein (gp133) | 3.36 | 19 | 1 | 1 | high | not found |
| 32. | 1. |  | *B. mycoides* | hypothetical protein | 0 | 1 | 1 | 1 | peak found | high |
| 33. | 2. |  | *B. mycoides* | 50S ribosomal protein L11 | 4.22 | 6 | 1 | 2 | peak found | not found |
| 34. | 3. |  | *B. mycoides* | **30S ribosomal protein S19** | **7.02** | **9** | **1** | **3** | **peak found** | **not found** |
| 35. | 4. |  | *B. mycoides* | hypothetical protein | 2.09 | 2 | 1 | 1 | not found | not found |
| 36. | 5. |  | *B. mycoides* | extracellular ribonuclease | 0 | 4 | 1 | 2 | high | high |
| 37. | 6. |  | *B. mycoides* | hypothetical protein | 0 | 8 | 1 | 2 | peak found | high |
| 38. | 7. |  | *B. mycoides* | hypothetical protein | 0 | 26 | 1 | 2 | peak found | not found |
| 39. | 8. |  | *B. mycoides* | ABC transporter permease protein YxdM | 0 | 7 | 1 | 1 | not found | not found |
| 40. | 1. |  | *Pseudomonas sp.* | hypothetical protein | 0 | 34 | 1 | 1 | high | not found |
| 41. | 2. |  | *Pseudomonas sp.* | hypothetical protein | 69.04 | 39 | 4 | 28 | high | not found |
| 42. | 3. |  | *Pseudomonas sp.* | hypothetical protein | 3.76 | 4 | 2 | 2 | high | not found |
| 43. | 4. |  | *Pseudomonas sp.* | hypothetical protein | 0 | 3 | 1 | 1 | high | not found |
| 44. | 5. |  | *Pseudomonas sp.* | hypothetical protein | 4.64 | 6 | 2 | 2 | high | peak found |
| 45. | 6. |  | *Pseudomonas sp.* | vitamin B12 transporter BtuB | 5.4 | 10 | 5 | 6 | high | peak found |
| 46. | 7. |  | *Pseudomonas sp.* | modulator of FtsH protease HflC | 6.28 | 17 | 5 | 6 | high | not found |
| 47. | 8. |  | *Pseudomonas sp.* | modulator of FtsH protease HflK | 4.54 | 7 | 2 | 3 | high | not found |
| 48. | 9. |  | *Pseudomonas sp.* | outer membrane protein TolC | 1.95 | 2 | 1 | 1 | high | not found |
| 49. | 10. |  | *Pseudomonas sp.* | lipid A export ATP-binding/permease protein MsbA | 0 | 3 | 1 | 1 | high | not found |
| 50 | 11. |  | *Pseudomonas sp.* | pyruvate dehydrogenase E1 component | 1.65 | 1 | 1 | 1 | high | not found |
| 51. | 12. |  | *Pseudomonas sp.* | sodium/proline symporter | 0 | 3 | 1 | 1 | high | not found |
| 52. | 13. |  | *Pseudomonas sp.* | 50S ribosomal protein L19 | 4.53 | 31 | 3 | 4 | high | not found |
| 53. | 14. |  | *Pseudomonas sp.* | penicillin-binding protein activator LpoA | 3.07 | 2 | 1 | 1 | high | not found |
| 54. | 15. |  | *Pseudomonas sp.* | ammonia monooxygenase gamma subunit | 4.86 | 10 | 2 | 2 | high | not found |
| 55. | 16. |  | *Pseudomonas sp.* | ubiquinol-cytochrome c reductase iron-sulfur subunit | 4.65 | 27 | 5 | 5 | high | not found |
| 56. | 17. |  | *Pseudomonas sp.* | hypothetical protein | 0 | 6 | 1 | 1 | high | not found |
| 57. | 18. |  | *Pseudomonas sp.* | hypothetical protein | 0 | 4 | 1 | 1 | high | not found |
| 58. | 19. |  | *Pseudomonas sp.* | hypothetical protein | 13.17 | 18 | 3 | 5 | high | peak found |
| 59. | 20. |  | *Pseudomonas sp.* | lipid A deacylase PagL | 2.48 | 10 | 1 | 1 | high | not found |
| 60. | 21. |  | *Pseudomonas sp.* | succinate dehydrogenase flavoprotein subunit | 8.16 | 9 | 3 | 4 | high | not found |
| 61. | 22. |  | *Pseudomonas sp.* | long-chain fatty acid transport protein | 15.84 | 19 | 5 | 7 | high | peak found |
| 62. | 23. |  | *Pseudomonas sp.* | FKBP-type 22 kDa peptidyl-prolyl cis-trans isomerase | 1.73 | 16 | 3 | 4 | high | not found |
| 63. | 24. |  | *Pseudomonas sp.* | hypothetical protein | 2.85 | 5 | 1 | 1 | high | not found |
| 64. | 25. |  | *Pseudomonas sp.* | **outer membrane porin F** | **177.47** | **43** | **11** | **46** | **high** | **peak found** |
| 65. | 26. |  | *Pseudomonas sp.* | multidrug/solvent efflux pump periplasmic linker protein MepA | 5.29 | 17 | 5 | 6 | high | peak found |
| 66. | 27. |  | *Pseudomonas sp.* | multidrug/solvent efflux pump membrane transporter MepB | 2.12 | 1 | 1 | 1 | high | not found |
| 67. | 28. |  | *Pseudomonas sp.* | leucine-, isoleucine-, valine-, threonine-, and alanine-binding protein | 0 | 3 | 1 | 1 | high | not found |
| 68. | 29. |  | *Pseudomonas sp.* | outer membrane lipoprotein SlyB | 5.37 | 15 | 1 | 2 | high | not found |
| 69. | 30. |  | *Pseudomonas sp.* | hypothetical protein | 1.95 | 8 | 1 | 1 | high | not found |
| 70. | 31. |  | *Pseudomonas sp.* | outer membrane protein assembly factor BamA | 2.42 | 4 | 3 | 3 | high | peak found |
| 71. | 32. |  | *Pseudomonas sp.* | esterase EstA | 0 | 3 | 1 | 1 | high | not found |
| 72. | 33. |  | *Pseudomonas sp.* | LPS-assembly protein LptD | 6.02 | 6 | 5 | 5 | high | peak found |
| 73. | 34. |  | *Pseudomonas sp.* | arylsulfatase | 1.96 | 4 | 2 | 2 | high | peak found |
| 74. | 35. |  | *Pseudomonas sp.* | Sec translocon accessory complex subunit YajC | 17.23 | 42 | 4 | 8 | high | peak found |
| 75. | 36. |  | *Pseudomonas sp.* | protein translocase subunit SecD | 6.3 | 6 | 3 | 3 | high | not found |
| 76. | 37. |  | *Pseudomonas sp.* | outer membrane protein assembly factor BamB | 4.79 | 10 | 2 | 4 | high | not found |
| 77. | 38. |  | *Pseudomonas sp.* | quinoprotein glucose dehydrogenase | 5.35 | 9 | 4 | 4 | high | not found |
| 78. | 39. |  | *Pseudomonas sp.* | porin B | 2.58 | 7 | 2 | 3 | high | not found |
| 79. | 40. |  | *Pseudomonas sp.* | flagellin | 35.14 | 35 | 5 | 12 | high | not found |
| 80. | 41. |  | *Pseudomonas sp.* | peptidyl-prolyl cis-trans isomerase D | 1.83 | 13 | 4 | 4 | high | not found |
| 81. | 42. |  | *Pseudomonas sp.* | FhuE receptor | 4.27 | 6 | 3 | 4 | high | peak found |
| 82. | 43. |  | *Pseudomonas sp.* | putative phospholipid ABC transporter-binding protein MlaD | 2.16 | 9 | 1 | 1 | high | not found |
| 83. | 44. |  | *Pseudomonas sp.* | cytochrome bo(3) ubiquinol oxidase subunit 2 | 8.78 | 10 | 2 | 3 | high | not found |
| 84. | 45. |  | *Pseudomonas sp.* | cytochrome bo(3) ubiquinol oxidase subunit 1 | 0 | 2 | 1 | 1 | high | not found |
| 85. | 46. |  | *Pseudomonas sp.* | N-substituted formamide deformylase | 8.89 | 4 | 1 | 6 | peak found | not found |
| 86. | 47. |  | *Pseudomonas sp.* | porin D | 2.18 | 6 | 2 | 2 | high | not found |
| 87. | 48. |  | *Pseudomonas sp.* | amino-acid carrier protein AlsT | 0 | 2 | 1 | 1 | high | not found |
| 88. | 49. |  | *Pseudomonas sp.* | ATP synthase subunit b | 20.63 | 26 | 4 | 9 | high | not found |
| 89. | 50 |  | *Pseudomonas sp.* | ATP synthase subunit c | 3.32 | 8 | 1 | 3 | high | not found |
| 90. | 51. |  | *Pseudomonas sp.* | outer membrane protein assembly factor BamD | 0 | 7 | 1 | 1 | high | not found |
| 91. | 52. |  | *Pseudomonas sp.* | porin D | 8.39 | 14 | 5 | 6 | high | not found |
| 92. | 53. |  | *Pseudomonas sp.* | hypothetical protein | 2.23 | 20 | 1 | 1 | high | not found |
| 93. | 54. |  | *Pseudomonas sp.* | LPS-assembly lipoprotein LptE | 6.26 | 13 | 2 | 3 | high | not found |
| 94. | 55. |  | *Pseudomonas sp.* | small-conductance mechanosensitive channel | 2.8 | 15 | 3 | 3 | high | not found |
| 95. | 56. |  | *Pseudomonas sp.* | porin D | 124.95 | 34 | 10 | 38 | high | peak found |
| 96. | 57. |  | *Pseudomonas sp.* | outer membrane protein P6 | 28.09 | 58 | 5 | 17 | high | not found |
| 97. | 58. |  | *Pseudomonas sp.* | outer membrane protein assembly factor BamD | 0 | 5 | 1 | 1 | high | not found |
| 98. | 59. |  | *Pseudomonas sp.* | vitamin B12 transporter BtuB | 10.76 | 8 | 6 | 7 | high | not found |
| 99. | 60. |  | *Pseudomonas sp.* | porin B | 0 | 8 | 3 | 3 | high | not found |
| 100. | 61. |  | *Pseudomonas sp.* | putative TonB-dependent receptor BfrD | 3.4 | 6 | 4 | 4 | high | not found |
| 101. | 62. |  | *Pseudomonas sp.* | ATP-dependent zinc metalloprotease FtsH | 0 | 2 | 1 | 1 | high | not found |
| 102. | 63. |  | *Pseudomonas sp.* | outer membrane protein assembly factor BamE | 0 | 12 | 1 | 1 | high | not found |
| 103. | 64. |  | *Pseudomonas sp.* | methyl-accepting chemotaxis protein McpS | 0 | 2 | 1 | 1 | high | not found |
| 104. | 65. |  | *Pseudomonas sp.* | glucans biosynthesis protein G | 1.85 | 2 | 1 | 1 | high | not found |
| 105. | 66. |  | *Pseudomonas sp.* | putative protein YibN | 2.59 | 9 | 1 | 1 | high | not found |
| 106. | 67. |  | *Pseudomonas sp.* | outer membrane protein W | 43.79 | 19 | 4 | 14 | high | not found |
| 107. | 68. |  | *Pseudomonas sp.* | bacterioferritin | 2.77 | 8 | 1 | 1 | high | not found |
| 108. | 69. |  | *Pseudomonas sp.* | 50S ribosomal protein L17 | 3 | 8 | 1 | 1 | not found | not found |
| 109. | 70. |  | *Pseudomonas sp.* | 30S ribosomal protein S4 | 4.98 | 4 | 1 | 2 | peak found | not found |
| 110. | 71. |  | *Pseudomonas sp.* | protein translocase subunit SecY | 0 | 2 | 1 | 1 | high | not found |
| 111. | 72. |  | *Pseudomonas sp.* | 30S ribosomal protein S5 | 3.3 | 5 | 1 | 2 | not found | not found |
| 112. | 73. |  | *Pseudomonas sp.* | 50S ribosomal protein L6 | 18.58 | 10 | 2 | 7 | peak found | not found |
| 113. | 74. |  | *Pseudomonas sp.* | 50S ribosomal protein L5 | 12.18 | 13 | 2 | 5 | high | not found |
| 114. | 75. |  | *Pseudomonas sp.* | 50S ribosomal protein L14 | 11.52 | 13 | 2 | 7 | peak found | not found |
| 115. | 76. |  | *Pseudomonas sp.* | 30S ribosomal protein S17 | 0 | 11 | 1 | 1 | high | not found |
| 116. | 77. |  | *Pseudomonas sp.* | 50S ribosomal protein L29 | 2.15 | 14 | 1 | 1 | peak found | not found |
| 117. | 78. |  | *Pseudomonas sp.* | 50S ribosomal protein L22 | 1.97 | 6 | 1 | 2 | peak found | not found |
| 118. | 79. |  | *Pseudomonas sp.* | 30S ribosomal protein S19 | 2.53 | 9 | 1 | 1 | peak found | not found |
| 119. | 80. |  | *Pseudomonas sp.* | 50S ribosomal protein L2 | 8.17 | 8 | 2 | 3 | not found | not found |
| 120. | 81. |  | *Pseudomonas sp.* | 50S ribosomal protein L23 | 6.09 | 18 | 1 | 3 | high | not found |
| 121. | 82. |  | *Pseudomonas sp.* | 50S ribosomal protein L4 | 2.26 | 5 | 1 | 2 | high | not found |
| 122. | 83. |  | *Pseudomonas sp.* | 50S ribosomal protein L3 | 2.44 | 9 | 2 | 2 | high | not found |
| 123. | 84. |  | *Pseudomonas sp.* | elongation factor Tu-A | 2.86 | 5 | 2 | 2 | high | not found |
| 124. | 85. |  | *Pseudomonas sp.* | Cbb3-type cytochrome c oxidase subunit CcoP1 | 3.72 | 2 | 1 | 2 | peak found | not found |
| 125. | 86. |  | *Pseudomonas sp.* | hypothetical protein | 7.02 | 13 | 2 | 4 | high | peak found |
| 126. | 87. |  | *Pseudomonas sp.* | Cbb3-type cytochrome c oxidase subunit CcoN1 | 1.96 | 2 | 1 | 1 | high | peak found |
| 127. | 88. |  | *Pseudomonas sp.* | ferripyoverdine receptor | 93.61 | 28 | 19 | 40 | high | peak found |
| 128. | 89. |  | *Pseudomonas sp.* | protein HemX | 0 | 9 | 2 | 2 | high | not found |
| 129. | 90. |  | *Pseudomonas sp.* | hypothetical protein | 0 | 4 | 1 | 1 | high | not found |
| 130. | 91. |  | *Pseudomonas sp.* | hypothetical protein | 0 | 15 | 1 | 1 | high | not found |
| 131. | 92. |  | *Pseudomonas sp.* | ferrichrome receptor FcuA | 1.64 | 8 | 4 | 4 | high | not found |
| 132. | 93. |  | *Pseudomonas sp.* | outer membrane efflux protein BepC | 0 | 3 | 1 | 1 | high | not found |
| 133. | 94. |  | *Pseudomonas sp.* | hypothetical protein | 0 | 8 | 1 | 1 | high | not found |
| 134. | 95. |  | *Pseudomonas sp.* | outer membrane efflux protein BepC | 1.89 | 4 | 2 | 2 | high | not found |
| 135. | 96. |  | *Pseudomonas sp.* | hypothetical protein | 2.38 | 5 | 1 | 1 | high | not found |
| 136. | 97. |  | *Pseudomonas sp.* | hypothetical protein | 1.81 | 6 | 2 | 2 | high | not found |
|  |  |  |  |  |  |  |  |  |  |  |

^9^, ^10^, ^11^

**References**

1. Meier-Kolthoff, J. P. & Göker, M. TYGS is an automated high-throughput platform for state-of-the-art genome-based taxonomy. *Nat. Commun.* **10**, 1–10 (2019).

2. Ondov, B. D. *et al.* Mash: Fast genome and metagenome distance estimation using MinHash. *Genome Biol.* **17**, 132 (2016).

3. Lagesen, K. *et al.* RNAmmer: consistent and rapid annotation of ribosomal RNA genes. *Nucleic Acids Res.* **35**, 3100–3108 (2007).

4. Camacho, C. *et al.* BLAST+: Architecture and applications. *BMC Bioinformatics* **10**, 421 (2009).

5. Meier-Kolthoff, J. P., Auch, A. F., Klenk, H. P. & Göker, M. Genome sequence-based species delimitation with confidence intervals and improved distance functions. *BMC Bioinformatics* **14**, 60 (2013).

6. Lefort, V., Desper, R., Gascuel, O. & Rosenberg, M. Brief Communication FastME 2.0: A Comprehensive, Accurate, and Fast Distance-Based Phylogeny Inference Program. *Mol. Biol. Evol* **32**, 2798–2800 (2798).

7. Farris, J. S. Estimating Phylogenetic Trees from Distance Matrices. *Am. Nat.* **106**, 645–668 (1972).

8. Kreft, Ł., Botzki, A., Coppens, F., Vandepoele, K. & Van Bel, M. PhyD3: a phylogenetic tree viewer with extended phyloXML support for functional genomics data visualization. doi:10.1093/bioinformatics/btx324

9. Tamura, K. Estimation of the number of nucleotide substitutions when there are strong transition-transversion and G+C-content biases. *Mol. Biol. Evol.* (1992). doi:10.1093/oxfordjournals.molbev.a040752

10. Kumar, S., Stecher, G. & Tamura, K. MEGA7: Molecular Evolutionary Genetics Analysis Version 7.0 for Bigger Datasets. *Mol Biol Evol* **33**, 1870–1874 (2016).

11. Kimura, M. A simple method for estimating evolutionary rates of base substitutions through comparative studies of nucleotide sequences. *J. Mol. Evol.* **16**, 111–120 (1980).
